# Quantitative TOF-MRA using SYNAPSE VINCENT predicts neointimal formation after stent-assisted coiling of intracranial aneurysms

**DOI:** 10.3389/fneur.2025.1723894

**Published:** 2026-01-13

**Authors:** Tohru Sano, Michiyasu Fuga, Issei Kan, Toshihiro Ishibashi, Naoki Kato, Gota Nagayama, Shunsuke Hataoka, Hiroyuki Enomoto, Yukiko Abe, Yuichi Murayama

**Affiliations:** 1 Department of Neurosurgery, Jikei University School of Medicine, Tokyo, Japan; 2 Department of Radiology, Jikei University Hospital, Tokyo, Japan

**Keywords:** artifact, cerebral aneurysm, complete occlusion, endovascular treatment, intracranial stent, neointimal formation, recanalization

## Abstract

**Introduction:**

Metallic susceptibility artifacts limit the reliability of time-of-flight MR angiography (TOF-MRA) for evaluating aneurysm healing after stent-assisted coiling (SAC). This study investigated whether quantitative TOF-MRA signal measurements at the aneurysm neck, analyzed using SYNAPSE VINCENT software, correlate with the angiographic white-collar sign (WCS), a surrogate marker of neointimal formation.

**Methods:**

Eighty-three internal carotid artery aneurysms in 81 patients treated with the Neuroform Atlas stent between January 2019 and December 2023 were retrospectively analyzed. Patients were categorized according to the presence or absence of WCS on 1-year digital subtraction angiography (DSA). The TOF-MRA signal intensity ratio (TOF-SIR) was defined as the ratio of signal intensity at the stented ICA neck to that at the distal M1 segment. TOF-SIR changes over 12 months were compared between groups. The primary outcome was the association between TOF-SIR change and WCS positivity; the secondary outcome evaluated longitudinal TOF-SIR trajectories within each group.

**Results:**

Of the 83 aneurysms, 39 (47%) demonstrated WCS positivity at 1 year. Complete occlusion rates were higher in the WCS-positive group immediately after SAC (23% vs. 11%, *p* = 0.031) and at 1 year (100% vs. 50%, *p* < 0.001). The increase in TOF-SIR from postoperative day 1 to 1 year was significantly greater among WCS-positive aneurysms (*p* = 0.004). ROC analysis identified a TOF-SIR change threshold of 0.03 as predictive of WCS positivity (sensitivity 80%; specificity 61%; AUC 0.69). After adjustment for confounders, TOF-SIR change remained independently associated with WCS development (OR 68.4; 95% CI 1.54–3,040). TOF-SIR increased progressively over time in WCS-positive aneurysms (*ρ* = 0.24, *p* = 0.002), whereas no significant longitudinal trend was observed in WCS-negative cases (*ρ* = 0.035, *p* = 0.65).

**Conclusion:**

A greater increase in TOF-SIR over 1 year was independently associated with WCS positivity, indicating that quantitative TOF-MRA using SYNAPSE VINCENT may serve as a robust surrogate marker of neointimal formation after SAC. This approach has the potential to reduce reliance on DSA and to function as a practical adjunct in long-term follow-up of aneurysm healing.

## Introduction

The development of low-profile intracranial stents has broadened the indications for stent-assisted coiling (SAC), particularly for wide-neck aneurysms (1). Although TOF-MRA is widely used for follow-up imaging, metallic stents frequently induce susceptibility artifacts that obscure visualization of the aneurysm neck (2). As a result, digital subtraction angiography (DSA) remains the gold standard for assessing aneurysm healing and recurrence, despite its invasiveness and radiation exposure (3–6).

Durable aneurysm occlusion after SAC depends on neointimal formation across the aneurysm neck (7). Histopathologic studies show progressive neointima covering stent struts (8), and experimental data indicate that MR susceptibility artifacts decrease over time as thrombus organizes and early neointimal tissue forms (9). These findings suggest that improved TOF-MRA visualization around the aneurysm neck may indirectly reflect neointimal formation, although this mechanism has not been demonstrated clinically.

Angiographically, neointimal coverage is often inferred from the “white-collar sign” (WCS)—a radiolucent gap between the coil mass and parent artery (10–12). Prior studies have shown that aneurysms with WCS have extremely low recurrence rates (0% vs. 6.8%) (13). Thus, establishing a relationship between TOF-MRA signal improvement and the presence of WCS may provide a non-invasive surrogate marker of neointimal formation.

To achieve more accurate assessment of vascular wall signals on TOF-MRA, we used SYNAPSE VINCENT software (Fujifilm, Tokyo, Japan), which applies vascular curved planar reformation (CPR) to straighten tortuous vessels. This straightened CPR display provides an intuitive depiction of the parent artery course and facilitates accurate visualization of the spatial relationship between the stented segment and adjacent downstream vessel segments, while allowing voxel-based signal analysis along the vascular centerline and reducing the influence of complex flow patterns and partial-volume effects (14). This approach enables more consistent wall-signal evaluation than conventional TOF-MRA and may improve the assessment of aneurysm occlusion and neointimal formation. Prior MRI studies have demonstrated that CPR enhances the reproducibility of quantitative vascular measurements by standardizing centerline-based sampling and reducing geometric overlap in tortuous segments (15), supporting the methodological validity of CPR-guided ROI placement in this study.

This study focused exclusively on aneurysms treated with the Neuroform Atlas stent (Stryker Neurovascular, Fremont, CA, United States) to minimize variability in susceptibility artifacts, which differ substantially across stent designs (16), and because its open-cell, low-profile architecture provides reliable wall apposition that may influence neointimal formation (17, 18). Restricting the analysis to a single device ensured methodological consistency for evaluating local MR signal characteristics. Accordingly, we examined whether TOF-MRA, combined with SYNAPSE VINCENT, can noninvasively assess aneurysm healing after SAC with the Neuroform Atlas stent. Specifically, we investigated the relationship between TOF-MRA signal intensity at the aneurysm neck and the angiographic WCS as an indirect marker of neointimal formation.

## Methods

### Study population

**Figure 1:**
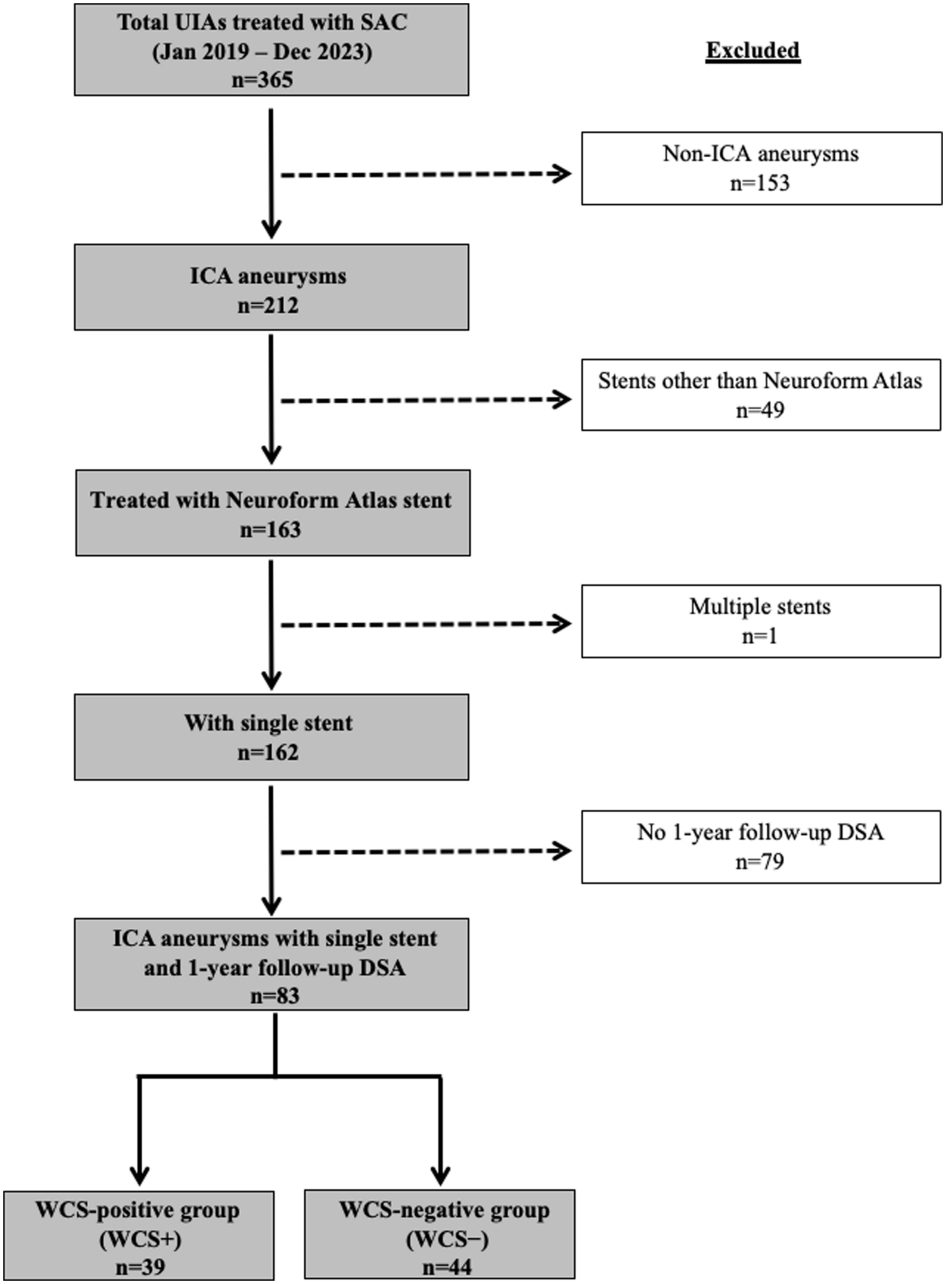
Flowchart of patient selection. Of 365 unruptured intracranial aneurysms (UIAs) treated with stent-assisted coiling (SAC) at The Jikei University Hospital between January 2019 and December 2023, 153 aneurysms located outside the ICA, 49 treated with stents other than the Neuroform Atlas, one case involving multiple stents, and 79 aneurysms without 1-year follow-up DSA were excluded. The final cohort consisted of 83 ICA aneurysms in 81 patients. Patients were classified into WCS-positive (WCS+) and WCS-negative (WCS–) groups based on the presence of the white-collar sign (WCS) on 1-year follow-up angiography.

A total of 365 consecutive unruptured intracranial aneurysms (UIAs) treated with SAC at our hospital between January 2019 and December 2023 were retrospectively identified from a prospectively maintained database. Of these, 153 lesions located outside the ICA and 49 treated with stents other than the Neuroform Atlas were excluded. One additional case involving multiple stents and 79 aneurysms without 1-year follow-up DSA were also excluded. Ultimately, 83 ICA aneurysms in 81 patients were included in the final analysis. Patients were categorized into two groups based on the presence or absence of the WCS on 1-year follow-up angiography: WCS-positive (WCS+) and WCS-negative (WCS−) (Figure 1).

This observational cohort study was conducted in accordance with the STROBE (Strengthening the Reporting of Observational Studies in Epidemiology) guidelines. Institutional review board approval was obtained, and the requirement for informed consent was waived owing to the retrospective study design.

### Data collection

Patient demographics, medical history, smoking and alcohol use, family history, aneurysm characteristics, procedural outcomes, and complications were obtained through retrospective review of medical records and radiological data. Aneurysm size and volume were measured using 3D-DSA. The Raymond-Roy Occlusion Classification (RROC) was used to evaluate occlusion status immediately post-procedure and at 1-year follow-up (19). The volume embolization ratio (VER) was calculated as follows: VER (%) = (volume of embolization coils/volume of aneurysm) × 100 (20). NeuroVision software (Cybernet Systems, Tokyo, Japan) was used for aneurysm and coil volume measurements (21). Recanalization was defined as an increase of at least one grade in the RROC or progression to Class III with an increase in the Meyers scale on follow-up DSA or MRA (22). Re-treatment was indicated if the RROC increased from Grade 1 or 2 to Grade 3, or if a Grade 3 aneurysm showed progression on the Meyers scale (23).

### Measurement of signal intensity at the aneurysm neck

TOF-MRA was performed using a 3.0-T MRI system (MAGNETOM Skyra 3 T, Siemens Healthineers, Erlangen, Germany) equipped with a 20-channel head and neck coil. Acquisition parameters were as follows: TR, 21.0 ms; TE, 3.7 ms; flip angle, 18°; voxel size, 0.3 × 0.3 × 0.5 mm; slice thickness, 0.5 mm; field of view, 210 mm; and acquisition time, 5 min 14 s. A multi-slab 3D TOF sequence with three slabs and an inferior saturation band was used.

**Figure 2:**
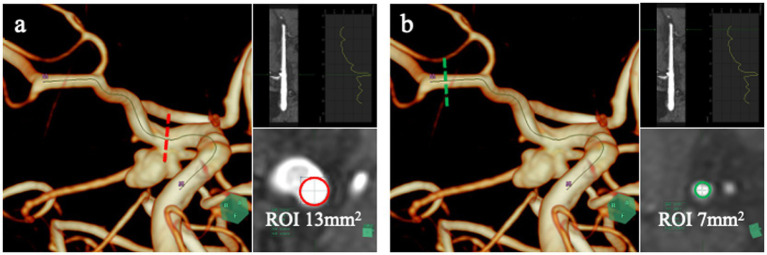
Quantitative TOF-MRA analysis using SYNAPSE VINCENT. SYNAPSE VINCENT was used to trace the vessel centerline from the ICA to the MCA and to extract signal-intensity values along this pathway. Signal intensity was assessed at two sites: a circular region of interest (ROI) placed in the ICA segment containing the stented aneurysm origin (**a**, red dotted line and red circle), and another ROI positioned in the distal M1 segment of the MCA (**b**, green dotted line and green circle), which served as the control. The M1 segment was chosen as the reference because it is unaffected by stent-related susceptibility artifacts and courses horizontally, enabling stable and reproducible signal-intensity measurements.

Signal-intensity analysis was performed using vascular CPR software (SYNAPSE VINCENT; Fujifilm). A vascular centerline was traced from the ICA to the MCA, and circular ROIs were placed along this pathway (Figure 2). Based on the vessel diameter in each case, the median ROI areas were 13.00 mm^2^ (IQR, 13.00–16.00 mm^2^) at the stented ICA aneurysm neck segment and 7.00 mm^2^ (IQR, 7.00–7.00 mm^2^) at the distal M1 segment. The ICA-neck ROI served as the target region for evaluating the stented aneurysm neck, whereas the distal M1 ROI served as the control because it is free of metallic artifacts and follows a stable horizontal course.

Two regions were therefore analyzed: (1) the stented ICA aneurysm neck and (2) the distal M1 segment of the MCA. The TOF-MRA signal intensity ratio (TOF-SIR) was calculated by dividing the signal intensity at the ICA neck by that at the M1 segment. TOF-SIR values were obtained at five time points: preoperatively, postoperative day 1, and at 3, 6, and 12 months. Changes in TOF-SIR over time were analyzed.

### Assessment of the white-collar sign

**Figure 3:**
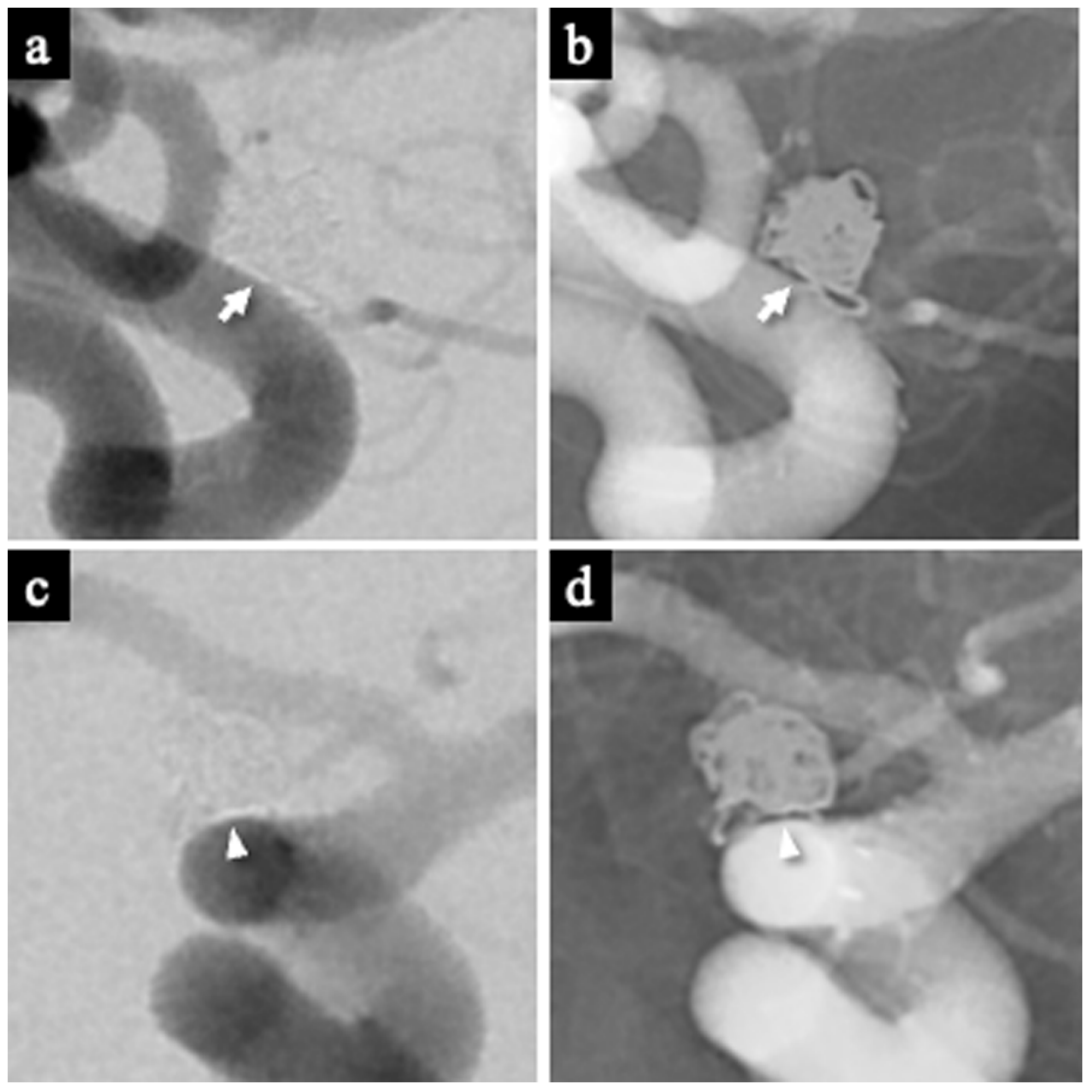
Representative images demonstrating the white-collar sign (WCS). **(a,b)** Left ICA aneurysm 1 year after coil embolization, showing a distinct interface between the aneurysm sac and the parent artery lumen. The WCS appears as a radiolucent gap (white arrow) on both DSA **(a)** and the corresponding nonsubtracted image **(b)**. **(c,d)** Left ICA aneurysm 1 year after coil embolization visualized on the down-the-barrel view. The WCS (white arrowhead) is identifiable on both DSA **(c)** and the corresponding nonsubtracted image **(d)**. WCS, White-Collar Sign.

The WCS was assessed on 1-year follow-up DSA. It was defined as a contrast-void, slit-like region extending across the aneurysm neck between the coil mass and the parent artery, visible on both subtracted and non-subtracted images (11). The optimal projection demonstrating the neck interface between the sac and parent artery was used for primary evaluation (Figures 3a,b), and this was supplemented by a down-the-barrel view when necessary (Figures 3c,d). Cases showing only partial non-filling zones were categorized as WCS-negative (12).

All angiographic examinations were performed using a biplane flat-panel detector system (Artis Q Biplane; Siemens Healthineers, Forchheim, Germany) with a 512 × 512 matrix and a 48-cm field of view. Images were reviewed on the syngo X-Workplace workstation (Siemens AG).

### Endovascular treatment

All SAC procedures were performed under general anesthesia by board-certified interventional neurosurgeons or under their direct supervision. Dual antiplatelet therapy (aspirin 100 mg/day and either clopidogrel 75 mg/day or prasugrel 3.75 mg/day) was initiated 1–4 weeks before the procedure. Intraoperatively, systemic heparinization was administered to maintain an activated clotting time >250 s. Postoperatively, argatroban or heparin was administered for 24 h to reduce thromboembolic risk. Dual antiplatelet therapy was continued for 6 months, followed by aspirin monotherapy for an additional 6 months.

### Outcomes

The primary outcome was the association between increases in TOF-SIR over time at the stented ICA aneurysm neck segment and WCS positivity, based on the hypothesis that greater TOF-SIR elevation reflects neointimal formation. The secondary outcome was the difference in longitudinal TOF-SIR changes between the WCS(+) and WCS(−) groups at the stented ICA segment.

### Interobserver agreement

TOF-SIR measurements and WCS assessments were independently performed by two board-certified interventional neurosurgeons (T. S. and M. F.), who were blinded to all clinical and angiographic outcomes. ROI placement followed a standardized protocol based on the vascular centerline and preoperative vessel diameter, thereby minimizing subjectivity in the measurement process. Discrepant cases were resolved through consensus with a third senior expert (I. K.). Prior MRI studies have shown that standardized manually placed ROIs provide good reproducibility in quantitative signal measurements (24), supporting the validity of our CPR-guided ROI approach.

### Statistical analysis

Continuous variables are presented as medians with interquartile ranges (IQRs) and were compared using the Mann–Whitney U test. Categorical variables were analyzed with Fisher’s exact test. Receiver-operating characteristic (ROC) curves were constructed to determine the optimal cutoff for TOF-SIR change between postoperative day 1 and 1 year in predicting WCS, using the Youden index (25).

Multivariate logistic regression analysis was then performed to evaluate whether this TOF-SIR change independently predicted WCS positivity. The model adjusted for variables that were significant in univariate analyses, as well as previously reported predictors of WCS: maximum aneurysm diameter, neck size, and VER (12, 13).

Spearman’s rank correlation coefficient was used to assess the association between TOF-SIR and time after treatment within each group. Statistical analyses were performed using R software, including EZR (Saitama Medical Center, Jichi Medical University, Saitama, Japan) (26). A *p* < 0.05 was considered significant.

## Results

### Patient characteristics

Of the 83 included unruptured ICA aneurysms, 39 (47%) were classified as WCS(+) based on 1-year follow-up DSA (Figure 1).

### Clinical and anatomical characteristics

Baseline characteristics are summarized in Table 1. There were no significant differences between the WCS(+) and WCS(−) groups in age, medical history (including hypertension, diabetes mellitus, dyslipidemia, polycystic kidney disease, and prior stroke), smoking or alcohol use, or family history. The proportion of female patients was significantly lower in the WCS(+) group compared with the WCS(−) group (62% vs. 86%, *p* = 0.012). No significant group differences were observed in aneurysm morphology, including maximum diameter, neck width, presence of blebs, or aneurysm location. All procedures were performed via the transfemoral approach.

**Table 1:** Clinical and anatomical characteristics and embolization outcomes in WCS-positive and WCS-negative groups.

**Variable**	**WCS positive *n* = 39**	**WCS negative *n* = 44**	***p*-value**
Clinical characteristics			
Age, years	54 [47, 66]	50 [46, 57]	0.1
Female sex	24 (62)	38 (86)	0.012*
Medical history			
Hypertension	12 (31)	12 (27)	0.81
Diabetes mellitus	2 (5.1)	1 (2.3)	0.6
Dyslipidemia	7 (18)	5 (11)	0.53
Polycystic kidney disease	2 (5.1)	2 (4.5)	1
Prior stroke	2 (5.1)	0 (0)	0.22
Smoking			0.19
Current	7 (18)	5 (11)	
Past	18 (46)	29 (66)	
Never	14 (36)	10 (23)	
Drinking	22 (56)	16 (36)	0.08
Family history of aneurysm	6 (15)	10 (23)	0.42
Anatomical characteristics			
Aneurysm characteristics			
Maximum diameter, mm	5.1 [4.4, 6.0]	5.2 [4.5, 5.9]	0.74
Neck, mm	4.1 [3.4, 4.9]	4.5 [3.9, 5.2]	0.2
Bleb formation	10 (26)	7 (16)	0.29
Aneurysm location			
ICA-SHA	11 (28)	18 (41)	0.17
ICA-OphtA	8 (21)	12 (27)	
ICA dorsal	8 (21)	2 (4.5)	
ICA-Pcom	7 (18)	9 (21)	
ICA-AChA	5 (13)	3 (6.8)	
Embolization outcomes			
Immediately after treatment			
RROC			
Class 1	9 (23)	5 (11)	0.031*
Class 2	24 (62)	21 (48)	
Class 3	6 (15)	18 (41)	
VER	28.0 [26.3, 32.0]	26.9 [24.1, 30.4]	0.18
Complications			
Ischaemic	0 (0)	1 (2.3)	1
Hemorrhagic	1 (2.6)	1 (2.3)	1
One year after treatment			<0.001*
RROC			
Class 1	39 (100)	22 (50)	
Class 2	0 (0)	19 (43)	
Class 3	0 (0)	3 (6.8)	
Recanalization	0 (0)	5 (11)	0.057
Retreatment	0 (0)	2 (4.5)	0.5
In-stent stenosis	1 (2.6)	2 (4.5)	1

AChA, anterior choroidal artery; ICA, internal carotid artery; OphtA, ophthalmic artery; Pcom, posterior communicating artery; RROC, Raymond-Roy occlusion classification; SHA, superior hypophyseal artery; VER, volume embolization ratio; WCS, white-collar sign. *p < 0.05. Unless otherwise indicated, values are presented as number of aneurysms (%) or as median [interquartile range]. Percentages may not total 100% due to rounding.

### Immediate embolization results and complications

Immediately after SAC, the distributions of RROC classifications differed between the two groups. In the WCS(+) group, 9 aneurysms (23%) were classified as RROC Class 1, 24 (62%) as Class 2, and 6 (15%) as Class 3. In the WCS(−) group, 5 aneurysms (11%) were classified as Class 1, 21 (48%) as Class 2, and 18 (41%) as Class 3. The rate of complete occlusion (RROC Class 1) was significantly higher in the WCS(+) group than in the WCS(−) group (*p* = 0.031). There was no significant difference in VER between groups. Similarly, the incidences of ischemic and hemorrhagic complications did not differ significantly between the groups (Table 1).

### Follow-up outcomes at 1 year

At 1-year follow-up, RROC outcomes differed markedly between groups. All 39 aneurysms (100%) in the WCS(+) group achieved complete occlusion (Class 1). In the WCS(−) group, 22 aneurysms (50%) were classified as Class 1, 19 (43%) as Class 2, and 3 (6.8%) as Class 3. Consequently, the complete occlusion rate was significantly higher in the WCS(+) group than in the WCS(−) group (*p* < 0.001).

The recanalization rate tended to be lower in the WCS(+) group (0%) than in the WCS(−) group (11%), although this difference did not reach statistical significance (*p* = 0.057). Re-treatment rates were comparable between groups. In-stent stenosis at 1 year occurred in 1 patient (2.6%) in the WCS(+) group and in 2 patients (4.5%) in the WCS(−) group, with no significant difference.

### Longitudinal TOF-SIR changes and WCS prediction

TOF-SIR values for the WCS(+) and WCS(−) groups are shown in Table 2. Median [IQR] values on postoperative day 1 were 0.50 [0.39–0.59] in the WCS(+) group and 0.56 [0.48–0.73] in the WCS(−) group. At 3 months, the values were 0.44 [0.38–0.63] and 0.50 [0.41–0.68], respectively; at 6 months, 0.53 [0.45–0.62] and 0.56 [0.43–0.72]; and at 1 year, 0.59 [0.50–0.69] and 0.58 [0.46–0.73]. No significant differences between groups were observed at any individual time point.

**Table 2:** TOF-SIR at the aneurysm neck within the stented segment.

Variable	WCS positive group *n* = 39	WCS negative group *n* = 44	*p*-value
Postoperative aneurysm neck/distal M1 TOF-SIR (median [IQR])			
1 day	0.50 [0.39, 0.59]	0.56 [0.48, 0.73]	0.05
3 month	0.44 [0.38, 0.63]	0.50 [0.41, 0.68]	0.19
6 month	0.53 [0.45, 0.62]	0.56 [0.43, 0.72]	0.54
1 year	0.59 [0.50, 0.69]	0.58 [0.46, 0.73]	0.95
Change in aneurysm neck/distal M1 TOF-SIR (median [IQR])^†^			
3 month	0.00 [−0.06, 0.06]	−0.02 [−0.11, 0.03]	0.38
6 month	0.03 [−0.03, 0.12]	0.01 [−0.07, 0.11]	0.26
1 year	0.08 [0.03, 0.19]	0.01 [−0.07, 0.11]	0.004*

TOF-SIR, time-of-flight signal intensity ratio. *p < 0.05. ^†^Postoperative day 1 signal intensity ratio is used as the reference value. Unless otherwise indicated, values are presented as median [interquartile range].

Changes in TOF-SIR relative to postoperative day 1 were then evaluated. From day 1 to 3 months, the change was 0.00 [−0.06 to 0.06] in the WCS(+) group and −0.02 [−0.11 to 0.03] in the WCS(−) group. From day 1 to 6 months, the change was 0.03 [−0.03 to 0.12] and 0.01 [−0.07 to 0.11], respectively. From day 1 to 1 year, the change was 0.08 [0.03 to 0.19] in the WCS(+) group and 0.01 [−0.07 to 0.11] in the WCS(−) group. Only the 1-year change differed significantly between groups (*p* = 0.004).

**Figure 4:**
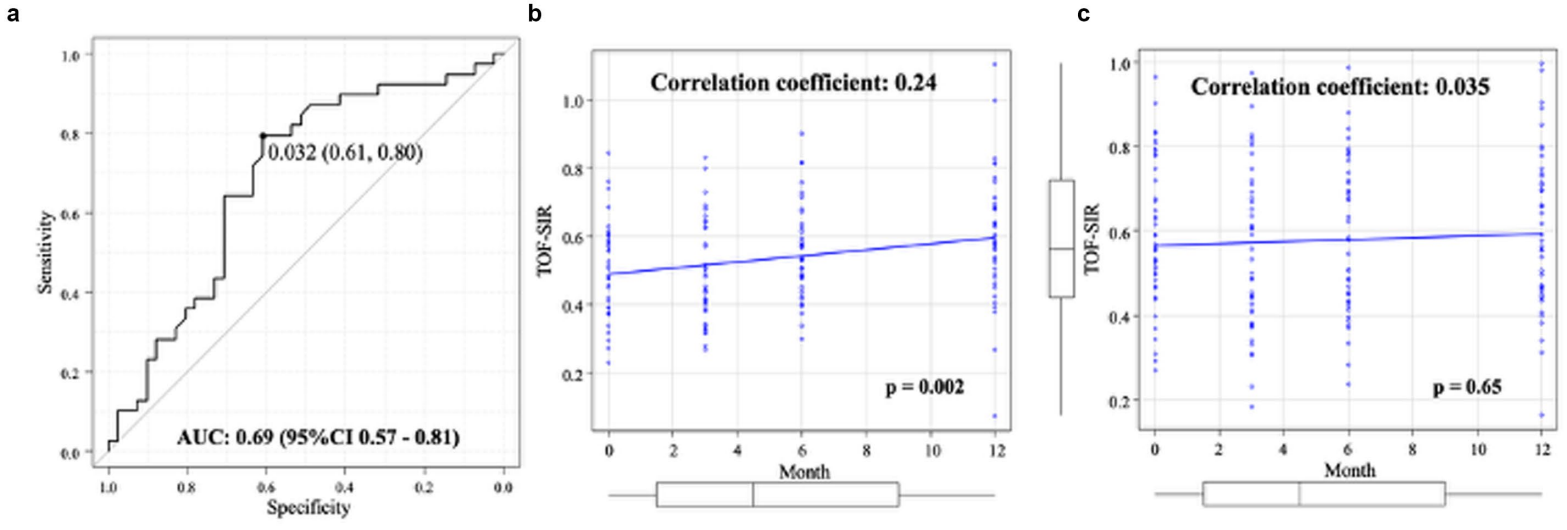
Quantitative evaluation of TOF-SIR changes. **(a)** ROC curve for determining the optimal cutoff value of TOF-SIR change from postoperative day 1 to 1 year in predicting WCS positivity. **(b)** In the WCS-positive group, TOF-SIR shows a significant positive correlation with time after treatment. **(c)** In the WCS-negative group, no significant correlation is observed between TOF-SIR and time after treatment. AUC, area under the curve; CI, confidence interval; ROC, receiver-operating characteristic; TOF-SIR, time-of-flight signal intensity ratio; WCS, white-collar sign.

ROC analysis identified a TOF-SIR change cutoff of 0.03 from postoperative day 1 to 1 year as predictive of WCS positivity, with a sensitivity of 80%, specificity of 61%, and an AUC of 0.69 (95% CI, 0.57–0.81) (Figure 4a). On multivariate logistic regression analysis, the TOF-SIR change from day 1 to 1 year independently predicted WCS positivity (OR 68.4; 95% CI, 1.54–3,040) (Table 3).

**Table 3:** Multivariate logistic regression analysis for predictors of WCS positivity.

Variables	OR (95% CI)	*p*-value
Female sex	0.17 (0.047 to 0.61)	0.006*
Maximum aneurysm diameter, mm	1.16 (0.66 to 2.04)	0.6
Aneurysm neck, mm	0.73 (0.41 to 1.28)	0.27
VER (%)	1.09 (0.99 to 1.19)	0.071
Change in aneurysm neck/distal M1 TOF-SIR from postoperative day 1 to 1 year^†^	68.4 (1.54 to 3,040)	0.029*

CI, confidence interval; OR, odds ratio; WCS, white-collar sign; VER, volume embolization ratio; TOF-SIR, time-of-flight signal intensity ratio. *p < 0.05. ^†^Postoperative day 1 TOF-SIR is used as the reference value.

In the WCS(+) group, a significant positive correlation was observed between TOF-SIR and time after treatment (*ρ* = 0.24, *p* = 0.002) (Figure 4b). In contrast, no significant correlation was found in the WCS(−) group (ρ = 0.035, *p* = 0.65) (Figure 4c).

### Representative cases

#### WCS-positive case

**Figure 5:**
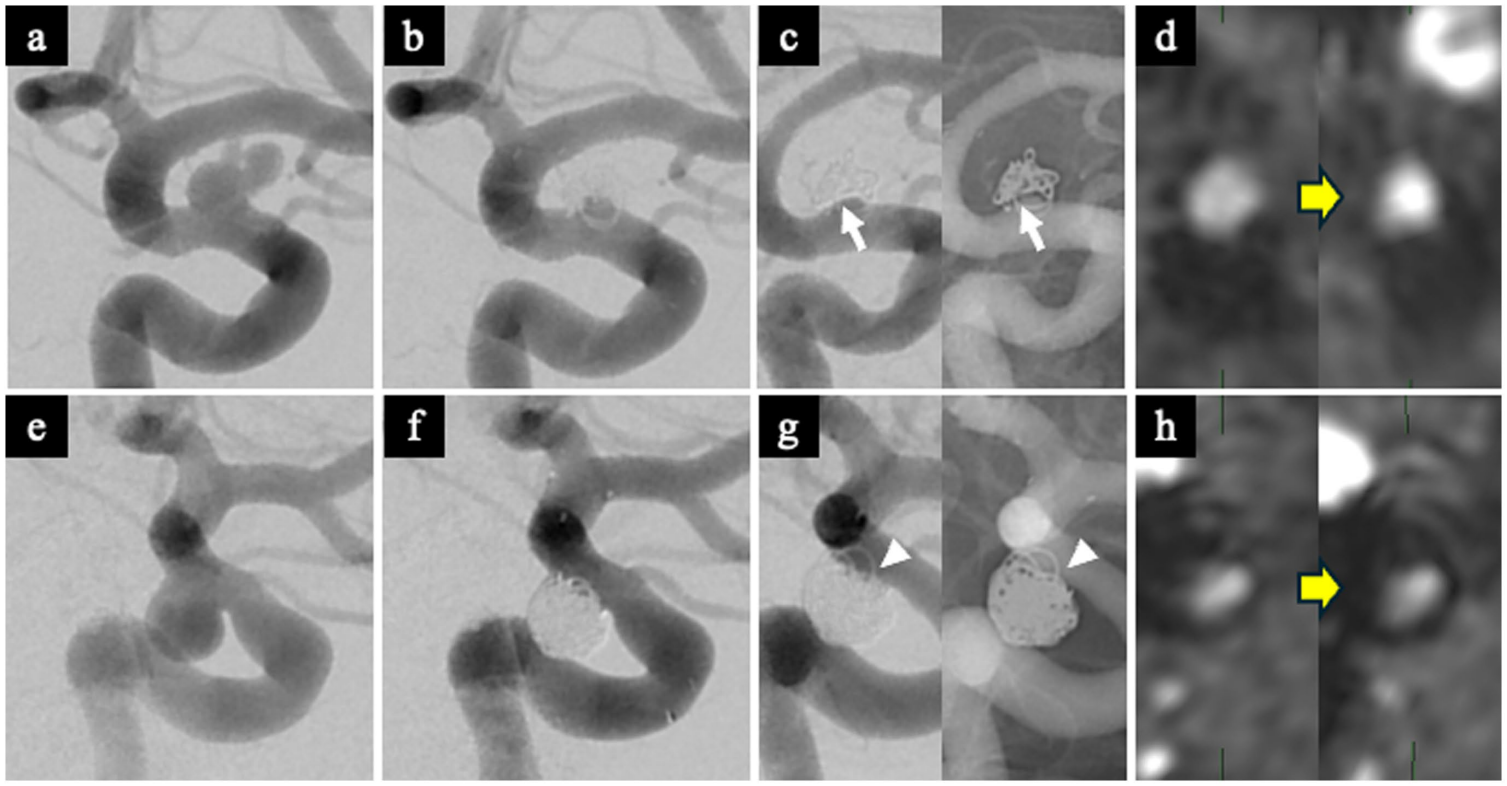
Representative WCS-positive and WCS-negative cases. WCS-positive case **(a–d)**. **(a)** Preoperative DSA showing a left ICA aneurysm. **(b)** Immediate post-embolization DSA demonstrating Raymond-Roy Occlusion Classification (RROC) Class 2 occlusion. **(c)** Follow-up DSA at 1 year showing a clear WCS at the aneurysm neck (white arrow). **(d)** Postoperative TOF-MRA showing increased TOF-SIR at 1 year compared with postoperative day 1, indicating improved vessel visualization. WCS-negative case **(e–h)**. **(e)** Preoperative DSA showing a left ICA aneurysm. **(f)** Immediate post-embolization DSA demonstrating RROC Class 2 occlusion. **(g)** Follow-up DSA at 1 year showing no evidence of the WCS (white arrowhead). **(h)** Postoperative TOF-MRA showing minimal change in TOF-SIR between postoperative day 1 and 1 year. RROC, Raymond-Roy Occlusion Classification; TOF-MRA, time-of-flight magnetic resonance angiography; TOF-SIR, time-of-flight signal intensity ratio; WCS, white-collar sign.

A 62-year-old man with a left ICA aneurysm (neck size: 4 mm) underwent SAC. The RROC classification immediately after treatment was Class 2, with a VER of 12.1% (Figures 5a,b). Follow-up DSA at 1 year clearly showed the WCS (Figure 5c). TOF-MRA showed an increase in TOF-SIR from 0.39 on postoperative day 1 to 0.60 at 1 year, corresponding to a change of 0.21 (Figure 5d).

#### WCS-negative case

A 38-year-old woman with a left ICA aneurysm (neck size: 4.6 mm) underwent SAC. The RROC classification was Class 2, and VER was 33.6% (Figures 5e,f). Follow-up DSA at 1 year did not demonstrate the WCS (Figure 5g). TOF-SIR showed minimal change, increasing from 0.29 on postoperative day 1 to 0.31 at 1 year, a change of only 0.02 (Figure 5h).

## Discussion

### Main findings

This study demonstrated that the change in TOF-SIR over the first year after treatment was significantly greater in the WCS(+) group than in the WCS(−) group. ROC analysis identified a TOF-SIR change threshold of 0.03 as predictive of WCS positivity. Notably, none of the WCS-positive aneurysms recurred (0/39), whereas 11% (5/44) of WCS-negative aneurysms showed recurrence, supporting the relevance of WCS as a marker of durable aneurysm healing. Multivariate logistic regression further confirmed that a larger TOF-SIR increase independently predicted WCS positivity (OR 68.4; 95% CI, 1.54–3,040), underscoring the utility of TOF-SIR change as a non-invasive radiological indicator of neointimal maturation.

A progressive increase in TOF-SIR was observed only in the WCS(+) group throughout the first year, consistent with the hypothesis that signal enhancement reflects neointimal growth across the aneurysm neck. A TOF-SIR increase of ≥0.03 may therefore serve as a practical indicator of neointimal coverage and a low likelihood of recurrence. These findings highlight the potential of TOF-MRA to non-invasively monitor aneurysm healing after SAC with the Neuroform Atlas stent and to reduce reliance on DSA, thereby minimizing procedural burden, cost, and radiation exposure.

Although Grüter et al. showed in a rat aneurysm model that neointimal formation rapidly becomes continuous by day 28 (8), vascular healing in humans progresses more slowly. Experimental and clinical studies indicate that endothelialization and neointimal maturation may remain incomplete for several months (27, 28). Early neointimal coverage (within the first 3–6 months) is therefore likely too thin to sufficiently attenuate susceptibility artifacts from stent struts, resulting in minimal TOF-SIR changes during this period. In contrast, further maturation and thickening of the neointima during longer-term healing lead to more prominent artifact reduction. This interpretation aligns with the steady TOF-SIR increase in WCS-positive cases and explains why a significant between-group difference emerged only at the 12-month follow-up.

### Challenges in imaging stented segments with MRI

MRI has demonstrated excellent accuracy in assessing aneurysm occlusion following coiling alone (29, 30). However, in SAC, metallic stents frequently introduce artifacts that obscure visualization of the parent artery and aneurysm neck, substantially limiting the diagnostic utility of MRI (4, 5, 31, 32). Consequently, DSA has remained the gold standard for post-treatment evaluation despite its invasiveness and associated risks (3–6, 33). Given these limitations, a reliable, non-invasive alternative for follow-up imaging has long been sought but remained unavailable in the context of SAC. The present findings suggest that TOF-SIR quantification using SYNAPSE VINCENT may serve as a practical, non-invasive adjunct to DSA for routine surveillance after SAC and, in selected patients, a potential non-invasive alternative in follow-up imaging.

### Advantages of TOF-MRA with SYNAPSE VINCENT

Accurate quantification of signal intensity at the vascular wall is essential for evaluating hemodynamic changes and vessel remodeling after endovascular treatment. However, conventional TOF-MRA is limited by flow-related signal loss, susceptibility artifacts, and difficulty isolating the signal from the stented segment. SYNAPSE VINCENT overcomes these limitations by applying vascular CPR, which straightens tortuous vessels along the centerline. This technique enables (1) more precise voxel-based analysis by reducing partial-volume effects, (2) fewer flow-related artifacts, and (3) more consistent sampling along the vascular wall. These capabilities enhance the reliability of TOF-MRA signal assessment and improve post-treatment vessel evaluation. In this study, SYNAPSE VINCENT facilitated objective quantification of TOF-SIR at the stented aneurysm neck, and its semi-automated, user-friendly workflow supports routine clinical implementation without requiring advanced technical expertise.

This study focused exclusively on aneurysms treated with the Neuroform Atlas stent to ensure consistent evaluation of TOF-MRA susceptibility artifacts. Artifact behavior varies markedly among intracranial stents owing to differences in strut geometry and device design, even when alloy composition is identical (16). The Neuroform Atlas features a highly conformable, low-profile, open-cell architecture with reliable wall apposition, as demonstrated in high-resolution imaging and clinical studies (17, 18). Because neointimal maturation depends in part on stent apposition, restricting analysis to a single stent platform minimized confounding and enabled a more controlled assessment of TOF-SIR as a marker of aneurysm healing.

### WCS as a surrogate marker of neointimal formation

To evaluate whether increases in TOF-SIR reflect neointimal growth, the WCS was used as an angiographic surrogate marker. Murayama et al. first demonstrated in an animal model that a radiolucent gap between the coil mass and the parent artery corresponds to neointima formation (10), and this radiolucent zone—now recognized as the WCS—has since been observed in human patients as well (11). In the present study, WCS positivity was strongly associated with favorable angiographic outcomes: all WCS-positive aneurysms achieved complete occlusion with no recurrences at 1 year, whereas only 50% of WCS-negative aneurysms achieved complete occlusion and 11% demonstrated recurrence. These findings align with previous reports indicating that the WCS reflects neointimal maturation and predicts durable aneurysm healing (10–13). Taken together, the evidence supports the WCS as a reliable angiographic indicator of neointimal formation.

### Limitations

Several limitations warrant consideration. First, no direct histological validation is available to confirm that elevated TOF-SIR reflects neointimal formation. Although WCS has been associated with neointimal coverage (10), further histopathological and imaging correlation studies are needed. Second, this study included only ICA aneurysms, which may limit generalizability to aneurysms in other locations, such as the posterior circulation. Third, all patients were treated with the Neuroform Atlas stent. Whether the present findings extend to other stent designs—such as the Low-profile Visualized Intraluminal Support (LVIS) stent (MicroVention Terumo, Tustin, CA) or the Enterprise stent (Cerenovus, Johnson & Johnson, New Brunswick, NJ)—remains uncertain. Fourth, this was a retrospective, single-center study with a moderate sample size. The wide confidence interval for the odds ratio of TOF-SIR change likely reflects the limited sample size and event distribution; thus, this result should be interpreted with caution. Despite these limitations, this study provides, to our knowledge, the first clinical evidence that increased TOF-SIR correlates with WCS positivity and may reflect neointimal development.

## Conclusion

A greater increase in TOF-SIR over 1 year was independently associated with WCS positivity, indicating that quantitative TOF-MRA using SYNAPSE VINCENT may serve as a robust surrogate marker of neointimal formation after SAC. This imaging-based biomarker has the potential to reduce reliance on DSA and function as a practical adjunct for routine surveillance, thereby minimizing procedural and radiation-related risks. In selected patients, quantitative TOF-MRA may also provide a viable alternative to DSA for long-term follow-up, although further validation is required.

## Data Availability

The raw data supporting the conclusions of this article will be made available by the authors, without undue reservation.
